# Healthcare workers’ experiences with integrated HIV and TB prevention in Liangshan, China: a qualitative exploration of barriers and enablers

**DOI:** 10.3389/fpubh.2025.1615781

**Published:** 2025-10-14

**Authors:** Ruili Bi, Rong Pei, Chunnong Jike, Gang Yu, Ju Wang, Zhonghong Wang, Yubin Wang, Xujia Zhang

**Affiliations:** ^1^School of Public Administration, China University of Geosciences, Wuhan, China; ^2^School of Public Health, Chengdu University of Traditional Chinese Medicine, Chengdu, China; ^3^Liangshan Prefecture Center for Disease Control and Prevention, Xichang, China

**Keywords:** HIV/TB integration, healthcare workers’ experiences, ethnic minority regions, qualitative study, health policy implementation

## Abstract

**Background:**

Evidence on frontline implementation of integrated HIV/TB prevention in resource-limited, ethnic minority regions remains limited. Liangshan Yi Autonomous Prefecture in Southwest China carries a dual HIV/TB burden. This study explored healthcare workers’ experiences with China’s Integrated Prevention and Control of Four Diseases (IPC4D) policy to identify barriers and enablers of service integration.

**Methods:**

A qualitative phenomenological study was conducted from July to December 2024. 37 semi-structured interviews were held with purposively sampled healthcare workers across prefectural CDCs, infectious disease hospitals, county hospitals, and township health centers. Interviews were audio-recorded, transcribed verbatim, and thematically analyzed following Braun and Clarke’s six-phase framework. Reflexive memos and triangulation across facility levels, professional roles, and ethnic groups enhanced study rigor.

**Results:**

Four themes emerged. First, policy-driven progress: participants reported greater governmental support, increased resource inputs, and modest improvements in public awareness. Second, structural barriers: chronic underfunding of TB services, workforce shortages, and burnout weakened integration. Third, the multi-sectoral “1 + M + N + P” model—local government leadership (“1”), township centers (“M”), village doctors and maternal–child health staff (“N”), and public security departments (“P”)—expanded service reach but also generated task overload, cultural–linguistic challenges, and inter-sectoral friction. Fourth, urban–rural divergence: township providers faced more severe infrastructure gaps and patient non-adherence, often driven by stigma and financial constraints.

**Conclusion:**

The IPC4D policy demonstrates potential to reduce HIV/TB disparities in Liangshan, yet sustained progress requires dedicated TB financing, culturally competent workforce training, rational task redistribution, and stigma-reduction strategies that leverage Yi community networks. These findings provide practical insights for adapting integrated disease-control policies in other high-burden, resource-constrained settings.

## 1 Introduction

Tuberculosis (TB) has reclaimed its position as the leading cause of death from a single infectious disease, with 10.8 million new cases and 1.25 million deaths globally in 2023 (1). HIV remains another persistent global health challenge, with approximately 39.9 million people living with HIV and 42.3 million cumulative deaths to date (2). TB is the most common opportunistic infection among people living with HIV (PLHIV), and continues to be the leading cause of HIV-related deaths (3). The dual burden of TB/HIV co-infection places significant strain on health systems, particularly in low- and middle-income countries, necessitating coordinated and integrated service delivery models (4, 5). In response, the World Health Organization (WHO) has issued a series of global guidelines advocating for integrated TB and HIV care. In 2012, WHO issued guidelines promoting collaborative TB/HIV activities, calling for joint planning, co-located services, and unified monitoring systems (6). In 2020, this agenda was expanded under the Global Framework for Multi-Disease Elimination, which emphasized the value of integrated, people-centered care models that address overlapping epidemics through coordinated prevention, treatment, and policy implementation (7). Integrated care models have since been shown to improve patient outcomes, streamline service delivery, and maximize the efficient use of constrained health resources (8, 9).

A growing body of literature demonstrates that integrated TB/HIV care improves patient outcomes, enhances resource use, and reduces service duplication (10, 11). However, implementation remains inconsistent in resource-limited settings, where systemic barriers—including inadequate health infrastructure, human resource constraints, and fragmented governance—continue to impede scale-up (12, 13). The COVID-19 pandemic further exposed the weaknesses of siloed vertical programs and renewed calls for integration to strengthen health system resilience (14, 15). China has achieved substantial progress in the control of HIV and TB epidemics; nevertheless, marked geographical inequities persist. Liangshan Yi Autonomous Prefecture—a remote, socio-economically disadvantaged region—continues to shoulder one of the heaviest HIV and TB burdens in the country, thereby exemplifying these persistent disparities (16, 17). As of September 2017, the prefecture had reported 35,513 people living with HIV, accounting for 4.7% of the national caseload (18). Concurrently, the notified TB incidence in Liangshan consistently exceeded 100 cases per 100,000 population between 2017 and 2021, registering a 6.62% increase in 2021 relative to 2017 (19). In response, the Chinese government implemented the “Integrated Prevention and Control of Four Diseases” (IPC4D) initiative in Liangshan in 2021 (20). This policy adopts a unified framework targeting HIV, TB, hepatitis C virus (HCV), and syphilis through integrated planning, coordinated service delivery, and shared performance metrics (21). The IPC4D framework underscores the synergies among these conditions, which share transmission routes, risk factors, and health system resources.

Preliminary evaluations suggest that IPC4D policy improves interdepartmental collaboration, promotes rational resource allocation, and enhances public awareness (22). However, the practical realities of implementing integrated care—particularly from the perspective of frontline healthcare workers (HCWs)—remain underexplored in China’s rural and ethnically diverse settings (23–25). While global evidence increasingly recognizes the role of HCWs in identifying barriers and informing service improvement, their voices are often underrepresented in Chinese IPC4D policy evaluations.

This study seeks to fill this gap by qualitatively examining healthcare workers’ (HCWs) experiences with delivering integrated HIV/TB services under the IPC4D framework in Liangshan. It focuses on exploring HCWs’ perceptions of the feasibility and effectiveness of IPC4D implementation, identifying structural, sociocultural, and operational barriers to service integration, and proposing contextually appropriate strategies to strengthen integrated disease control in underserved settings.

By centering HCWs’ experiential knowledge, this research contributes critical insights into the challenges and opportunities of service integration in under-resourced contexts. The findings have implications not only for China’s ongoing efforts toward integrated disease control but also for global strategies seeking to improve health equity through coordinated, locally responsive care models.

## 2 Materials and methods

### 2.1 Study design and setting

This qualitative study employed a phenomenological approach to explore healthcare workers’ lived experiences in implementing the IPC4D policy in Liangshan Yi Autonomous Prefecture, Southwest China—a region with an HIV prevalence exceeding 1% in several counties and TB incidence rates nearly double the national average (26). The study was conducted between July and December 2024 in a range of healthcare settings, including the Liangshan Prefectural CDC, infectious disease hospitals, county-level hospitals, and township health centers.

### 2.2 Research team and reflexivity

The study team comprised three investigators with complementary expertise. Interviews were conducted by the first author (RLB), a female Ph.D candidate in public administration with three years of qualitative field experience at CDCs and primary-care facilities in Southwest China, and the second author (RP), a public-health researcher experienced in health-system integration and research with marginalized populations. Neither had prior contact with participants, reducing social-desirability bias. CJ, a senior administrator at Liangshan CDC and an ethnic-Yi physician with >20 years of HIV/TB prevention experience, facilitated site access, staff engagement, and cultural-linguistic guidance. Additionally, two independent qualitative consultants contributed to interview design and protocol development.

To enhance reflexivity and trustworthiness, the research team held regular reflective meetings during data collection and analysis to identify and address potential biases, such as interviewer positionality and assumptions. Reflexive memos were kept to track evolving interpretations and decisions. Analytical rigor was strengthened through triangulation across facility levels, professional roles, and geographic regions. Although participants could review transcripts for factual accuracy upon request, full member checking of thematic analysis was not performed due to logistical constraints, which is acknowledged as a study limitation.

### 2.3 Participant selection and recruitment

A total of 37 healthcare workers were recruited using purposive sampling strategies designed to ensure diversity in gender, age, professional role, years of experience, and ethnic background. Eligible participants included front-line doctors and nurses, township-based HIV and TB prevention workers, maternal and child health staff, hospital administrators, infection control specialists, and county-level CDC directors. Inclusion was based on active involvement in HIV and/or TB prevention and control activities under the IPC4D policy. Participants were either recommended by the interview sites or recruited through telephone contact, and all participated voluntarily. No individuals withdrew from or declined to participate in the interviews.

### 2.4 Informed consent and ethical considerations

Written informed consent was obtained from all participants prior to data collection. The consent process included a detailed explanation of the study’s purpose, voluntary participation, study procedures, and measures to ensure confidentiality and anonymity. The study protocol was reviewed and approved by the Medical Ethics Committee of the Affiliated Hospital of Chengdu University of Traditional Chinese Medicine (Approval No. 2023KL-099). No incentives were provided. All data were de-identified to protect participant identities.

### 2.5 Data collection

**Figure 1:**
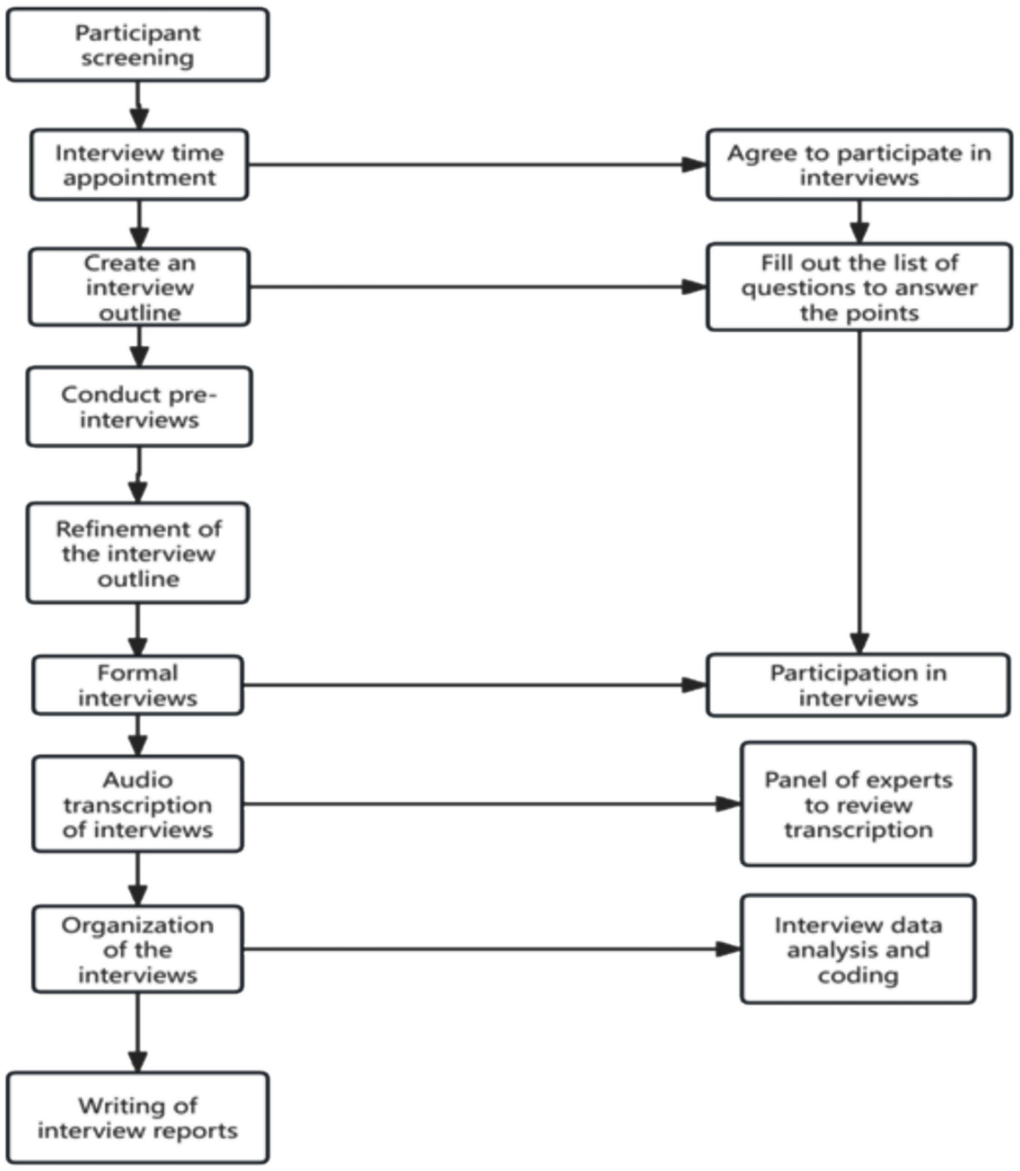
Flow-chart of recruitment and inclusion of healthcare workers.

In-depth, semi-structured interviews were conducted face-to-face, in private rooms or offices at participants’ workplaces according to their preference. The guide, informed by the HIV/TB integration literature and refined after two pilot interviews with non-participating CDC staff, covered post-IPC4D prevention changes, intersectoral collaboration, adherence, resource allocation and institutional challenges. Interviews (30–45 min) were audio-recorded with consent; post-interview field notes captured non-verbal cues and contextual observations. No repeat interviews were performed. Transcripts were returned on request for clarification, but participant validation of findings was not undertaken owing to logistical constraints. Figure 1 shows the flow of this health workers interview.

### 2.6 Data analysis

Thematic analysis followed Braun and Clarke’s six-phase approach (27), with coding and theme development conducted using NVivo 12 software. The process consisted of four key steps:

1. Familiarization with Data: The first author listened to all recordings and read transcripts multiple times to become immersed in the data.
2. Inductive Coding: Three members of the research team independently performed line-by-line open coding on a subset of transcripts. Codes were generated inductively, without predefined categories.
3. Theme Development and Refinement: After independent coding, the team held consensus meetings to reconcile coding discrepancies and discuss emerging categories. Disagreements were resolved through iterative discussion. No external coders were involved.
4. Mapping and Framework Alignment: The final themes were mapped to relevant WHO guidance on integrated care for TB and HIV to facilitate contextual interpretation.

Themes were considered finalized when no new concepts emerged during the last five interviews, indicating data saturation. A simplified coding tree was developed to illustrate how raw textual data were translated into codes and thematic categories (Supplementary Appendix 1). Illustrative quotes were selected to reflect thematic diversity and are labeled with anonymized participant IDs indicating professional role and ethnic background.

### 2.7 Research team roles

The research team comprised eight members with complementary roles. RLB, a doctoral candidate in public administration, led fieldwork, conducted interviews, performed primary coding, and drafted the manuscript. RP, an experienced qualitative public health researcher, co-conducted interviews, contributed to study design, and engaged in reflexive analysis. CJ, a senior staff member at the Liangshan CDC, facilitated recruitment, field access, and provided cultural insights. GY and JW managed audio recording, note-taking, and transcript organization, while ZHW, YBW, and XJZ undertook verbatim transcription and initial coding under senior guidance. All authors contributed to theme development, analytical refinement, and manuscript revision to ensure coherence and credibility.

## 3 Results

### 3.1 Participant characteristics

This study included 37 healthcare workers (HCWs) from Liangshan Yi Autonomous Prefecture, selected through purposive sampling to ensure variation in gender, age, ethnicity, education, professional role, and years of experience. Participants included front-line HIV and TB prevention workers, township and county-level CDC staff, maternal and child health professionals, hospital infection control personnel, and administrators. Their professional experience ranged from under 1 year to nearly 40 years.

The sample included 20 females and 17 males. Participants identified as Yi (*n =* 18), Han (*n* = 18), and Tibetan (*n* = 1), reflecting the local ethnic composition. Educational backgrounds varied from secondary technical school to bachelor’s degrees, with majors in clinical medicine, nursing, laboratory science, and public health. A wide range of positions were represented, including directors of CDCs and hospitals, township health center managers, village doctors, and ART clinic staff. Table 1 presents the detailed demographic and professional information of all participants, including their ID, sex, age, ethnicity, education, major, years in current role, and professional title.

**Table 1:** Baseline characteristics of the study participants (*n* = 37).

Id	Sex	Age (years)	Ethnicity	Education level	Major	Years in current position	Professional role
P1	Female	34	Yi	Associate degree	Clinical medicine	6	HIV prevention worker
P2	Male	44	Yi	Bachelor	Health management	8	Director, county CDC
P3	Male	36	Han	Bachelor	Clinical medicine	2	Deputy director, County CDC
P4	Male	42	Yi	Associate degree	Clinical medicine	0.8	Director, county CDC
P5	Male	44	Yi	Bachelor	Clinical medicine	5	Head of HIV/AIDS prevention and control section
P6	Male	35	Han	Bachelor	Clinical medicine	3	Head of HIV/AIDS prevention and control section
P7	Female	48	Yi	Associate degree	Nursing	8	Head of HIV/AIDS prevention and control section
P8	Female	31	Han	Bachelor	Clinical medicine	1	Deputy director, ART center, county hospital
P9	Female	37	Yi	Bachelor	Clinical medicine	10	Director, ART center, county hospital
P10	Female	42	Yi	Bachelor	Nursing	6	Deputy director, county health bureau
P11	Male	41	Yi	Secondary Technical School	Community medicine	6	Director, township health center
P12	Female	28	Han	Bachelor	Nursing	1	HIV prevention worker
P13	Female	33	Han	Associate degree	Pharmacy	3	HIV prevention worker
P14	Female	40	Han	Associate degree	Preventive medicine	13	HIV prevention worker
P15	Female	22	Yi	Associate degree	Nursing	3	HIV prevention worker
P16	Female	26	Tibetan	Associate degree	Laboratory medicine	2	HIV prevention worker
P17	Male	41	Yi	Associate degree	Clinical medicine	2	Deputy director, township health center
P18	Male	45	Yi	Bachelor	Clinical medicine	2	Director, township health center
P19	Male	50	Han	Associate degree	Clinical medicine	13	Director, county CDC
P20	Female	46	Han	Associate degree	Clinical medicine	25	Village doctor
P21	Male	47	Yi	Associate degree	Clinical medicine	25	Village doctor
P22	Male	36	Yi	Associate degree	Nursing	4	HIV prevention worker
P23	Male	32	Yi	Bachelor	Clinical medicine	5	Head of HIV/AIDS prevention and control section
P24	Female	45	Han	Bachelor	Clinical medicine	22	Director, infectious disease hospital
P25	Male	60	Han	Bachelor	Infectious diseases	39	Former head, infectious disease department
P26	Female	49	Han	Bachelor	Infectious diseases	20	Head, infectious disease department
P27	Male	38	Han	Bachelor	Infectious diseases	10	Attending physician, infectious disease department
P28	Male	33	Han	Bachelor	Preventive medicine	6	Deputy director, STD and HIV center
P29	Male	29	Han	Bachelor	Preventive medicine	3	HIV prevention worker
P30	Female	27	Han	Bachelor	Medical laboratory science	3	Tuberculosis control worker
P31	Female	28	Yi	Bachelor	Clinical laboratory science	4	Tuberculosis control worker
P32	Male	42	Han	Associate degree	Integrative chinese and western medicine	6	HIV prevention worker
P33	Female	39	Han	Bachelor	Clinical medicine	1	Tuberculosis control worker
P34	Female	36	Yi	Associate degree	Clinical medicine	14	Tuberculosis control worker
P35	Female	38	Yi	Associate degree	Clinical medicine	15	Maternal and child health worker
P36	Female	37	Yi	Bachelor	Clinical medicine	6	Maternal and child health worker
P37	Female	45	Han	Associate degree	Clinical medicine	9	Maternal and child health worker


Data collected from March to June 2024. CDC, center for disease control; ART, antiretroviral therapy.

### 3.2 Themes and sub-themes

During the thorough process of data analysis, several significant themes and concepts emerged. To ensure a comprehensive understanding of this extensive data set, we have decided to examine the analysis through the perspective of these key themes. Table 2 presents the four primary themes identified in the analysis. Below, we summarize these themes and include illustrative quotes from healthcare workers.

**Table 2:** Themes and sub-themes constituting the research findings, Liangshan, China. 2024.

Themes	Sub-themes
Theme 1: Policy-driven progress in HIV/TB integration	(1) Increased perception of government support (2) Perceived improvements in resource allocation (3) Mixed views on the effectiveness of public awareness campaigns
Theme 2: Current status of HIV-TB integrated prevention and control	(1) Routine TB screening among PLHIV (2) Dual management of HIV/TB Co-infections (3) Challenges in managing drug-resistant TB
Theme 3: Perceived strengths and Limitations of the “1 + M + N + P” Model	(1) Local government (“1”): leading and coordinating resources (2) Township health centers (“M”): technical backbone of the system (3) Village doctors, HIV prevention workers, and maternal-child health workers (“N”): overburdened community frontliners (4) Involvement of grassroots police (“P”) and challenges of collaboration
Theme 4: Implementation challenges	(1) Workforce shortages and burnout (2) Funding gaps and imbalances (3) Patient non-adherence and mistrust

#### 3.2.1 Theme 1: Policy-driven progress in HIV/TB integration

##### 3.2.1.1 Increased perception of government support

Participants widely reported that since HIV/AIDS and tuberculosis (TB) were jointly incorporated into the IPC4D policy, local governments have markedly increased their prioritization of these two major infectious diseases. The policy explicitly mandates that Party secretaries at the prefecture, county, township, and village levels (“four-level” leadership) take direct responsibility for infectious disease control, ensuring political commitment and administrative support from the top down. Under this governance framework, medical institutions have strengthened their prevention and control efforts, while government departments provide continuous oversight and accountability.

“In Liangshan, both HIV/AIDS and TB prevalence rank among the highest in the country. Therefore, the Party committees and government have always centered their prevention and control work on HIV/AIDS, which has historically been the focal point of intensive control efforts.” (P5, male, Yi, Head of HIV/AIDS Prevention and Control Section).

Through government-led mobilization, annual universal HIV screening has been institutionalized, with population-based screening conducted according to household registration to maximize case detection. For migrant workers living outside the county, the policy requires two viral load tests per year (once in each half-year) and facilitates cross-regional coordination, including reimbursement for travel expenses, to reduce testing barriers.

“Migrant workers can undergo testing at local hospitals in their work location, then send back the test report, ID card, and a photo of themselves holding both. For example, if a patient is working in Shanghai, the government may reimburse round-trip travel costs and arrange free testing.” (P11, male, Yi, Director, Township Health Center).

Compared with HIV prevention, universal TB screening was initiated later. Since 2023, it has been implemented in high-priority counties, while screening of student populations began in 2021—the same year IPC4D was launched—given the high risk of transmission in densely populated school settings.

“We mainly conduct universal TB screening in the ten priority counties of Aila. So far, we have completed about 60%–70% of the population screening. All students must be screened because schools are densely populated areas and must be prioritized.” (P30, female, Han, Tuberculosis control worker).

Several participants emphasized that integrating HIV and TB into the IPC4D framework, alongside a scientifically designed performance assessment system, has effectively promoted screening, testing, case tracing, and follow-up. By breaking down performance targets to specific townships, the approach has created a goal-oriented mechanism that has yielded significant improvements.

“Previously, without an assessment mechanism, many tasks could not be carried out. Now, to meet assessment targets, we allocate each target to every township. No matter what approach is taken, the targets must be met, and so far the results have been clear.” (P33, female, Han, Tuberculosis control worker).

##### 3.2.1.2 Perceived improvements in resource allocation

Several participants noted that following the implementation of the IPC4D policy, their healthcare institutions integrated HIV and tuberculosis (TB) prevention and control into a single department. This restructuring allowed for unified allocation of human and material resources, thereby reducing duplication and waste. Within this integrated framework, the well-established HIV prevention and control system was extended to TB management, enabling coordinated control of both diseases at the grassroots level. While HIV prevention remains the primary focus, the level of attention to TB has increased markedly.

“Previously, in single-disease prevention, both policy attention and the concentration of medical resources were primarily focused on HIV. Since the implementation of the IPC4D policy, TB has received more attention than before.” (P2, male, Yi, Director, County CDC).

Frontline TB control staff, in particular, reported perceiving substantial benefits from the policy. Although some operational details remain to be refined, the inclusion of TB within the “four diseases” joint prevention and control framework has enabled TB programs to leverage the existing HIV network, monitoring systems, and management practices, while also securing greater support from higher-level authorities.

“Since 2021, our TB department has essentially been riding on the ‘giant ship’ of HIV prevention to carry out our work. But I think what is most important is that the higher authorities have increased their attention to TB.” (P34, female, Yi, tuberculosis control worker).

This resource consolidation approach has not only enhanced the efficiency and coverage of HIV and TB prevention and control but has also provided a practical model for integrated multi-disease control in grassroots healthcare institutions operating under resource constraints.

##### 3.2.1.3 Mixed views on the effectiveness of public awareness campaigns

Participants reported a noticeable increase in public awareness of the four major infectious diseases, which they attributed to targeted educational campaigns implemented under the IPC4D policy. With strengthened governmental support, public outreach efforts have become more systematic, and medical staff have received training to enhance communication with the public. These efforts were perceived to have improved treatment adherence—particularly among newly diagnosed patients—and increased participation in nationwide screening initiatives. Enhanced awareness has also fostered greater public engagement in health examinations and better cooperation in early detection and treatment.

“Since the implementation of the IPC4D policy, at least now, whether it is staff at various levels of medical institutions, including grassroots units, or ordinary residents, everyone knows—more or less—that HIV and TB are infectious diseases harmful to health. Public awareness of such diseases has increased significantly.” (P25, male, Han, former head, Infectious Disease Department).

The importance of culturally sensitive public education campaigns was emphasized, particularly for raising awareness about prevention. Several participants highlighted the value of integrating health promotion into local cultural events—such as the Yi Torch Festival and Yi New Year—to maximize community engagement. For school-aged children, participants underscored the significance of involving them in health education, noting that children often become effective conduits of information to their families. This approach, referred to as *“Little Hands Holding Big Hands,”* was seen as particularly impactful.

“One lesson from a child to their parents is more effective than many lessons from us. So we utilized school health classes to teach the students about HIV and TB, and through the students, we could convey this knowledge to the parents.” (P1, female, Yi, HIV prevention worker).

Home visits were also identified as an essential means of reinforcing disease awareness, with healthcare workers regularly visiting households to provide information and distribute free condoms. In remote areas where regular outreach is challenging, designated health promotion days are organized to deliver essential knowledge on major infectious diseases such as HIV and TB.

#### 3.2.2 Theme 2: Current status of AIDS and TB integrated prevention and control

##### 3.2.2.1 Routine TB screening among people living with HIV

Tuberculosis (TB) remains one of the most common comorbidities among people living with HIV (PLHIV). Most respondents emphasized that due to the weakened immunity of PLHIV, they are at substantially higher risk of TB infection. Consequently, TB screening is routinely conducted alongside HIV treatment. Once HIV–TB co-infection is confirmed, patient information is promptly shared with TB control personnel to facilitate timely follow-up, home visits, and treatment adherence monitoring.

“This year, our hospital admitted two patients with both HIV and TB, both of whom were diagnosed with TB during HIV treatment. TB status in such cases is reassessed every six to nine months. Once TB is detected, we share the patient information with TB control staff so they can better track the patient’s condition.” (P9, Female, Yi, Director of the ART Center, County Hospital).

Respondents also reported that TB patients are, in principle, required to undergo HIV testing annually. However, in practice, challenges remain. Some patients complete HIV testing at designated hospitals but fail to return their reports for system entry, while some health facilities have yet to implement HIV screening for TB patients. These gaps can delay the detection of co-infections. The implementation of the IPC4D Policy has therefore played a pivotal role in advancing HIV–TB integrated prevention and control, including the unified management of co-infected patients.

“Now, TB and HIV are managed within the same department under the same leadership, which greatly facilitates the management of HIV–TB co-infected patients. We also recommend that primary healthcare facilities adopt this model, unlike before when HIV and TB control were handled entirely separately.” (P8, Female, Han, Deputy Director of the ART Center, County Hospital).

##### 3.2.2.2 Dual management of HIV/TB co-infections

According to tuberculosis control workers, the implementation of the IPC4D Policy has transformed the management of HIV and TB from two separate, disease-specific programs to a coordinated dual-management approach, especially for patients co-infected with both HIV and TB. In this model, both diseases are treated simultaneously, ensuring comprehensive care and follow-up. One staff member from the Liangshan CDC explained that monthly epidemiological analyses now involve cross-referencing the HIV and TB databases to identify cases of co-infection. These patients are then managed jointly by both the HIV and TB teams.

“For HIV–TB co-infected patients under our care, the TB department also keeps their records and conducts follow-up and adherence monitoring. If privacy concerns prevent them from contacting a patient directly, we step in to coordinate communication. We never manage HIV alone—both departments work together and support each other.” (P1, Male, Yi, HIV prevention worker).

Healthcare providers involved in tuberculosis (TB) treatment have observed that some patients co-infected with HIV and TB refuse to attend follow-up appointments due to concerns about privacy breaches. In these situations, staff specializing in HIV prevention often take the lead in educating and counseling patients, as they tend to be more trusted and are generally more experienced in handling this population. Any TB-related information gathered by HIV prevention workers is then shared with TB clinicians.

“For example, one patient refused all telephone follow-up due to fears of disclosure. The HIV prevention team handled all contact, education, and management, and later shared the TB-related information with the TB treatment physician.” (P26, Female, Han, Head of the Infectious Disease Department).

Respondents emphasized that the treatment of HIV-TB coinfection should prioritize the patient’s most prominent symptoms. For patients experiencing acute respiratory symptoms caused by TB, treatment for TB is initiated immediately, while antiretroviral therapy (ART) is generally started about two weeks later, in accordance with clinical guidelines. If any drug–drug interactions arise, alternative medications are chosen to ensure that both treatment regimens can continue effectively.

“There are now many advanced treatment options for HIV–TB co-infections, and they generally do not conflict. If a conflict does arise, we simply adjust the regimen. There are plenty of alternatives for both HIV and TB.” (P27, Male, Han, Attending Physician, Infectious Disease Department).

##### 3.2.2.3 Challenges in managing drug-resistant TB

Many participants highlighted the challenges of managing drug-resistant infections, noting that drug-resistant TB was far more common than drug-resistant HIV. They attributed this to the high TB burden in Liangshan, limited diagnostic capacity, and the concentration of cases among Yi ethnic communities, where communal living accelerates transmission. Low education levels contributed to poor treatment adherence, and many DR-TB patients were co-infected with HIV, often neglecting treatment for both diseases, resulting in immunosuppression and heightened vulnerability to drug-resistant TB.

“Liangshan’s drug-resistant TB rate is among the highest nationally, with a current rate of approximately 18%. This is primarily due to non-adherence to medication regimens, with a smaller proportion resulting from primary resistance to drug-resistant TB strains.” (P24, female, Han, director, Infectious Disease Hospital).

To address the needs of drug-resistant TB patients, the designated infectious disease hospital in Liangshan Prefecture has established a dedicated “green channel” for direct online coordination with other hospitals. This pathway ensures priority admission and reserved beds for drug-resistant TB patients, while also providing lower-tier facilities with specialized guidance and technical support.

“As long as a drug-resistant TB patient requires hospitalization, we prioritize them through the green channel and try our best to help lower-level hospitals resolve any treatment or technical difficulties.” (P25, male, Han, former head, Infectious Disease Department).

Another challenge frequently mentioned was the financial burden associated with drug-resistant TB treatment. While first-line anti-TB drugs are provided free of charge and 80% of DR-TB treatment costs can be reimbursed through medical insurance, the cost of second-line anti-TB drugs remains high, making it difficult for some patients to afford these essential medications.

#### 3.2.3 Theme 3: The innovative “1 + M + N + P” model for grassroots infectious disease prevention and control

Since its implementation in 2017 in Liangshan Prefecture, the “1 + M + N + P” model has aimed to decentralize major infectious disease prevention efforts, particularly for HIV and tuberculosis (TB). The goal is to enhance multisectoral coordination and improve coverage and precision at the grassroots level. This model consists of four key components: “1” refers to local government leadership and coordination, “M” represents township health centers that provide technical support, “N” includes village doctors, HIV prevention workers, and maternal and child health workers responsible for conducting routine follow-ups and community health education, and “P” consists of grassroots police officers who support epidemiological investigations and patient tracing efforts (28). While most participants acknowledged the positive outcomes of this model, many also noted structural challenges and role conflicts during its implementation.

##### 3.2.3.1 Local government (“1”): leading and coordinating resources

Many interviewees highlighted the crucial role of local governments in mobilizing resources, enforcing policies, and managing mobile populations—particularly for patients with poor adherence or high mobility.

“When township HIV prevention workers and village doctors fail to trace individuals or encounter non-adherent cases, government intervention becomes necessary. But confidentiality must be respected, so informed consent is required.” (P7, Female, Yi, Head of HIV/AIDS Prevention and Control Section).

“Problems like self-relocated households cannot be solved at our level. The government must set unified rules—for example, requiring medical checkups before individuals are allowed to travel.” (P32, Male, Han, HIV prevention worker).

##### 3.2.3.2 Township health Centers (“M”): technical backbone of the system

As the core technical units at the grassroots level, township health centers are responsible for case registration, medical follow-up, and basic care. In high-burden counties, they have taken on expanded responsibilities, including antiretroviral treatment (ART).

“In priority counties, responsibilities like follow-up, medication supervision, and health promotion have been delegated to township health centers. Some are even administering ART.” (P28, Male, Han, Deputy Director, STD & HIV Center).

However, participants from non-integrated institutions reported a lack of resources or role inclusion:

“Our hospital wasn’t given funding or personnel support. These cases are managed like chronic diseases, not treated as special conditions.” (P24, Female, Han, Director, Infectious Disease Hospital).

##### 3.2.3.3 Village doctors, HIV prevention workers, and maternal-child health workers (“N”): overburdened community Frontliners

The group referred to as “N” includes frontline implementers who are responsible for home visits, ART adherence monitoring, maternal-child health interventions, and health education. Their role is essential in connecting individuals with the healthcare system, particularly in remote areas. However, respondents have reported widespread understaffing and an overwhelming workload due to the IPC4D policy.

“We used to handle only HIV. Now, with the ‘four-disease co-prevention’ policy, we have to manage multiple diseases, do community education, and conduct home visits.” (P10, Female, Yi, Deputy Director, County Health Bureau).

“We conduct monthly home monitoring for pregnant women who are HIV-positive. Two recent cases have resulted in successful Prevention of Mother-To-Child Transmission (PMTCT) outcomes, but the workload is heavy and detailed.” (P13, Female, Han, HIV prevention worker).

##### 3.2.3.4 Involvement of grassroots police (“P”) and challenges of collaboration

Grassroots police were incorporated to assist with contact tracing and the management of high-risk populations. However, ambiguities in their roles, limited interdepartmental communication, and lack of financial incentives have constrained their effectiveness.

“Police involvement sometimes provokes resistance. It’s better if the township government takes the lead.” (P21, Male, Yi, Village doctor).

“Public security doesn’t coordinate directly with health services, so their role is limited. They should receive proper funding; without it, no one wants to take on risky tasks.” (P32, Male, Han, HIV prevention worker).

Some participants also noted the limited applicability of the model in low-prevalence areas:

“In counties with very few cases, township involvement isn’t necessary—county-level staff can handle everything.” (P28, Male, Han, Deputy Director, STD & HIV Center).

#### 3.2.4 Theme 4: Implementation challenges

##### 3.2.4.1 Workforce shortages and burnout

Most participants reported a markedly increased workload and heightened stress following the integration of HIV and TB prevention into the “IPC4D” policy. Whereas staff previously managed HIV or TB as single diseases, they are now required to address four infectious diseases simultaneously—HIV, TB, hepatitis C, and syphilis. In many facilities, the same teams that once handled single-disease control are now responsible for multi-disease prevention, inevitably resulting in a substantial surge in workload.

“Previously, one department only needed to handle HIV prevention, but now we manage four or more diseases, including follow-up, supervision, and household visits. Orders are issued by party and government authorities and passed down through all levels of the health system, placing enormous pressure on frontline staff.” (P19, Male, Yi, Director, Township Health Center).

This situation is especially common in primary care facilities. Respondents from township health centers noted that liaison with county CDC staff is only part of their duties. They must also provide treatment and follow-up for HIV and TB, manage mother-to-child transmission (MTCT) prevention for HIV, hepatitis C, and syphilis, conduct population-wide health screenings, and compile extensive data reports.

“Primary healthcare workers face heavy workloads, and high staff turnover means that individuals often take on multiple roles. At the same time, higher-level authorities have strict requirements for disease data management, demanding more written records and supporting documentation, which creates significant stress.” (P18, Male, Yi, Director, Township Health Center).

Maternal and child health workers similarly described being stretched thin. In addition to MTCT prevention, they are tasked with multi-disease management, household visits, chronic disease care, and services for impoverished populations, with repetitive and overlapping responsibilities that lead to exhaustion.

“Besides the three diseases in MTCT prevention, I also handle health check-ups, chronic disease management, and maternal care. One person is responsible for all public health work—it can be overwhelming at times.” (P35, Female, Yi, Maternal & Child Health Worker).

##### 3.2.4.2 Funding gaps and imbalances

A recurring concern among participants was the lack of dedicated funding for TB prevention and control. Although both HIV and TB have been included in the “IPC4D” policy, TB was perceived as receiving less attention and fewer resources compared to HIV. National funding allocations are disproportionately focused on HIV programs, creating significant shortfalls for TB-related activities—especially for drug-resistant TB. As a result, many patients are forced to discontinue treatment due to the heavy financial burden.

“The cost of treating drug-resistant TB is already very high, and many patients with limited financial means simply cannot afford it. This year we secured a subsidy of 100,000 RMB, which we distributed to 30 patients, giving each 3,000 RMB. But this is far from enough to cover even a single treatment regimen for a drug-resistant TB case.” (P9, Female, Yi, Director of the ART Center, County Hospital).

Participants highlighted that while first-line anti-TB drugs are available at no cost, TB screening usually requires chest CT scans, and in some instances, several diagnostic tests are necessary to confirm the disease. These diagnostic expenses are not entirely covered by medical insurance, forcing patients to pay out of pocket.

“TB is highly contagious. When patients come to the hospital, we recommend chest radiography for further examination, but many refuse due to the associated costs. This makes our work very challenging. We hope for increased funding to support TB diagnostics.” (P8, Female, Han, Deputy Director of the ART Center, County Hospital).

In addition to the lack of patient subsidies for TB, respondents also called for greater financial support for TB control activities. Some noted that even without funding, prevention tasks are still expected to be completed, though with limited effectiveness. The imbalance in financial incentives was also cited as a factor affecting staff engagement.

“Most of the funding is dedicated to HIV prevention and control, with substantial rewards for HIV prevention staff conducting follow-up and adherence supervision. However, incentives for TB control workers are relatively low, which reduces their motivation.” (P4, Male, Yi, Director of the County CDC).

##### 3.2.4.3 Patient non-adherence and mistrust

Most participants highlighted the ongoing challenges in ensuring patient adherence to treatment. Specifically, some newly diagnosed HIV patients identified through universal screening were hesitant to cooperate due to concerns about privacy breaches. These patients often refused home visits from healthcare workers and, in some instances, even issued verbal threats. Disrupting the chain of transmission is crucial for controlling HIV; however, during contact tracing, many patients deliberately conceal their sources of infection, which impedes epidemiological investigations.

“Some patients contracted HIV through commercial sex but conceal the fact for fear that family or friends might find out. In some cases, older men acquired the infection from street-based sex workers. Under such circumstances, tracing the source is almost impossible.” (P14, Female, Han, HIV prevention worker).

Maternal and child health workers also reported difficulties when pregnant women and their partners were uncooperative or frequently away from home. Strategies to address these issues included mobilization through township governments and community leaders, face-to-face health education, home visits during holiday periods, and encouraging partner testing during antenatal visits.

“Some pregnant women and their partners are unwilling to cooperate, especially those working away from home. We try to keep in touch via village doctors or HIV prevention workers, and when they return during holidays, we visit them to provide health education. We also encourage them to bring their partners for free HIV testing when they attend antenatal care.” (P36, Female, Yi, Maternal & Child Health worker).

In TB prevention and control, the main barrier to adherence is patients’ limited awareness of the disease, often associated with low educational levels. This lack of understanding leads some patients to stop treatment prematurely, despite receiving repeated counseling.

“Even if you tell them a hundred times not to stop the medication, they will not listen. Some patients treat it like a cold—once they feel better, they stop taking the drugs. Even patients treated for seven or eight years behave this way, unable to understand why they must continue treatment after symptoms have resolved.” (P33, Female, Han, Tuberculosis control worker).

## 4 Discussion

This study systematically examined the experiences and perceptions of healthcare workers across different administrative levels and professional backgrounds regarding integrated HIV and tuberculosis prevention and control under the IPC4D policy in Liangshan Prefecture. Findings revealed encouraging advances driven by the policy, innovative explorations of inter-sectoral collaboration at the primary-care level, and persistent challenges related to workforce capacity, financial resources, patient adherence, and cultural adaptation. Beyond addressing a critical evidence gap concerning frontline implementation of multi-disease integrated control in ethnic-minority regions of China, these results provide actionable insights for policy and practice in other resource-constrained, high-burden settings.

Participants generally perceived that since HIV and TB were incorporated into the IPC4D policy, government attention and support have markedly increased, with primary healthcare institutions gaining greater access to policy, financial, and technical resources. This finding aligns with evidence from low- and middle-income countries such as Uganda and South Africa (29, 30), where integrated HIV/TB services have been shown to facilitate resource mobilization, improve early diagnosis, and enhance treatment adherence. However, our study further revealed that in practice, funding was disproportionately allocated, with HIV programs receiving relatively sufficient support while tuberculosis—particularly drug-resistant TB—remained underfunded. This imbalance led some patients to discontinue treatment due to financial constraints, thereby undermining the overall effectiveness of integration. Similar challenges have been widely documented globally, and both WHO and UNAIDS have repeatedly emphasized that TB remains the leading cause of death among people living with HIV, underscoring the urgent need for more equitable allocation of resources within integrated HIV/TB programs (31, 32).

The grassroots “1 + M + N + P” model was regarded as an important innovation in integrated HIV/TB control. It combines the organizational leadership of local Party committees and governments (“1”), the technical support provided by township health centers (“M”), the frontline implementation carried out by village doctors, HIV prevention workers, and maternal and child health staff (“N”), and the collaboration of local public security departments (“P”) in contact tracing and follow-up activities. This multi-sectoral collaboration is particularly critical in remote areas, as it expands service coverage and improves case detection. Comparative evidence from other high-burden settings, such as Zambia and Tanzania, similarly demonstrates that multi-sectoral approaches can enhance service accessibility and strengthen community engagement (33–35). Nevertheless, our study revealed notable differences in how this model was perceived across professional roles: managers emphasized organizational coordination and policy implementation, while frontline staff highlighted the challenges of task overload and hidden work burden. In addition, in remote ethnic minority regions, cultural and language barriers further constrained implementation efficiency. These findings suggest that future scale-up of the model should prioritize task optimization and culturally sensitive communication strategies.

Most participants reported that the integration of HIV and TB services under the IPC4D framework has led to task diversification and increased workload. This challenge was particularly evident in rural and remote areas, where healthcare workers were often required to simultaneously manage HIV, TB, prevention of mother-to-child transmission, and chronic disease care, but lacked systematic training and professional support. These finding echoes prior evidence that task integration can contribute to professional burnout (36–38). Our study further revealed that maternal and child health staff as well as village doctors frequently conducted household follow-ups during holidays or when migrant populations returned home, relying on trust-building through informal, friend-like communication. While such efforts enhanced the effectiveness of disease control, in the absence of adequate training, incentives, and psychosocial support, sustained work overload may compromise service quality and undermine workforce retention.

Poor patient adherence was repeatedly emphasized by participants. Some newly diagnosed individuals living with HIV refused follow-up visits due to concerns about confidentiality, and in certain cases even verbally threatened outreach workers. Patients with drug-resistant TB often discontinued treatment because of financial hardship. In Yi ethnic minority communities, limited awareness of the necessity of long-term medication, coupled with shame and stigma related to sexual behaviors, led some patients to conceal their infection history, thereby impeding contact tracing. These findings are consistent with international evidence showing that stigma and low health literacy undermine the effectiveness of integrated HIV/TB control efforts in rural and minority populations (39–41). Future interventions should incorporate culturally sensitive health education and community mobilization strategies, leveraging the influence of local opinion leaders to strengthen adherence.

Aligned with global evidence, our findings corroborate the advantages of HIV/TB collaborative activities articulated by WHO and UNAIDS: integrated models shorten treatment delays, optimise resource utilization, and enhance service accessibility. Compared with South Africa’s widely cited “one-stop” model (42), Liangshan’s 1 + M + N + P framework demonstrates superior multi-sectoral engagement and outreach to remote populations, yet remains suboptimal with respect to resource equity, frontline human-resource security, and cultural adaptation. Both the WHO End TB Strategy and the Framework for Global Health 2050 underscore the imperative of people-centred integrated services (43, 44); our results extend this discourse by indicating that the success of integrated prevention and control in ethnic-minority regions ultimately hinges on the quality of cross-sector collaboration, the strength of community trust, and the rationalisation of task burdens.

Notably, perceptions of integration varied across different groups. In terms of professional roles, managers and frontline healthcare workers held divergent priorities. Ethnically, cultural and language barriers were more pronounced in Yi ethnic regions. Although age and gender were not systematically analyzed in this study, some younger female healthcare providers placed greater emphasis on issues such as patient stigma and personal safety. These findings suggest that future research should conduct stratified comparisons of experiences across different subgroups to better inform the design of tailored support strategies.

This study focuses on an integration model that encompasses not only the coordinated diagnosis and treatment of patients with HIV/TB co-infection, but also the broader collaboration between the two disease programs at the primary care level. The implementation of IPC4D policy in Liangshan has been ongoing for over three years, and this study was conducted two years after its initial rollout; thus, the experiences captured reflect the mid-phase implementation of the strategy. Although HIV programs have received sufficient investment, inadequate funding for TB—particularly drug-resistant TB programs—highlights imbalances in the execution of IPC4D across different components and administrative levels. This underscores the need to better define the scope and depth of integration.

The integration examined in this study extends beyond the clinical management of HIV/TB co-infected patients to encompass the broader coordination of the two disease control programs at the primary care level. In Liangshan, the IPC4D policy has been in place for more than three years, and this study was conducted approximately two years after its initiation, thereby reflecting the mid-term stage of implementation. While HIV programs have received relatively sufficient investment, funding for TB—particularly drug-resistant TB—remains inadequate. This imbalance indicates that the implementation of IPC4D policy has not been uniform across components and levels of care, underscoring the need to further clarify the scope and depth of integration.

## 5 Limitations

This study employed a qualitative design, with participants representing diverse professional roles and ethnic backgrounds; however, patient perspectives were not included, and member-checking was not conducted, which may introduce researcher interpretation bias. In addition, most respondents were recruited from public healthcare institutions, with limited representation from the private sector, thereby constraining the generalizability of the findings. Although differences across professional roles were analyzed, variations by gender and age were not systematically examined. Future research should integrate secondary epidemiological data with patient experiences and adopt multi-site, stratified designs to provide a more comprehensive understanding of the effectiveness and challenges of integrated strategies across different populations.

## Data Availability

The original contributions presented in the study are included in the article/Supplementary material, further inquiries can be directed to the corresponding author/s.
